# Characterization of the Retinal Circulation of the Mouse

**DOI:** 10.1167/iovs.65.14.3

**Published:** 2024-12-02

**Authors:** Fei Shang,, Jesse Schallek

**Affiliations:** 1 Department of Neuroscience, University of Rochester, Rochester, NY, United States; 2 Center for Visual Science, University of Rochester, Rochester, NY, United States; 3 Flaum Eye Institute, University of Rochester, Rochester, NY, United States

**Keywords:** retinal vasculature, transgenic animals, mouse, retinal imaging

## Abstract

**Purpose:**

Mice are highly used in retinal research because, like humans, mice have vascularized retinas and choroidal circulation. Although the retinal circulation has been well-characterized in development, its stability during adulthood is less understood. To examine this network, we quantified several key metrics of the trilaminar vasculature.

**Methods:**

We used mice (*n* = 15) with transgenic fluorescent NG2-DsRed (JX: #00824), a vascular-associated label in the retina. One eye per mouse was imaged using confocal microscopy (Nikon A1 Ti2 Eclipse) and traced with ImageJ SNT tools. Using an adaptive optics scanning light ophthalmoscope, additional mice (*n* = 3) were imaged at single-cell resolution within the living eye to measure the same vasculature.

**Results:**

Across mice, we found a stable retinal circulation that formed and maintained a trilaminar stratification throughout early adulthood at all eccentricities. Bridging these layers, microvessels had five distinct anatomical branching patterns. The superficial, intermediate, and deep plexuses increased in density with depth: 16.14 ± 3.61 mm/mm^2^, 22.14 ± 6.86 mm/mm^2^, and 31.01 ± 6.24 mm/mm^2^, respectively. This patterning was not impacted by eccentricity or age (13–61 weeks). Similar metrics were achieved using adaptive optics scanning light ophthalmoscope in vivo with the same analysis pipeline.

**Conclusions:**

The mouse retinal vasculature was stable up to 50 weeks of age, providing a robust and extensive baseline dataset with which models of retinal vascular and neural disease may be compared. Vessels connecting the laminae were more complex than previously reported and represented a uniquely vulnerable population due to their relatively low density.

Mice are the most used mammalian model for retinal research and have elucidated our understanding of the anatomy,^1^^,^^2^ development,^3^ and physiological properties of the eye. The murine retina shares many characteristics with the human retina, such as a similar organization of neurons, glia, and vasculature, making it popular for translational testing and applications. For example, diabetic retinopathy and glaucoma models have revealed vascular phenotypes such as acellular capillaries, pericyte loss, leaky vessels, and arteriole diameter changes^4^^–^^10^ that replicate aspects of human disease.

The ocular circulation is divided into two blood supplies: the choroidal and the retinal circulation. The first is responsible primarily for the deep layers of the outer retina, and the latter is responsible primarily for the inner retina.^11^^,^^12^ The mouse retinal circulation is further divided into the superficial, intermediate, and deep plexuses located in the nerve fiber layer and ganglion cell layer (GCL), inner plexiform layer, and outer plexiform layer,^13^^,^^14^ respectively. A complete three-dimensional analysis is important, because vascular coverage, length, and degree of branching are skewed when averaged together into a singular imaging plane.^14^

In the current study we visualize the vascular network by using transgenic mice that demonstrate bright, long-lasting neuron-glial antigen 2 (NG2) fluorescence throughout adulthood.^15^ We chose this model for two reasons. One, its background is C57BL/6J, the most common mouse strain used in research. Two, it avoids histological complications such as tissue penetration and protein depolymerization^16^ while providing a highly specific vascular label that enables identification of arterioles, venules, and pericytes based on morphological appearance.^15^^,^^17^

Despite the mouse model's prevalence and ability to recapitulate aspects of human disease, there are limited ground-truth retinal vascular datasets across the range of eccentricities in healthy adult mice. Therefore, we imaged from the central axis of the eye (near the optic disc)^2^ to the edge of the retina at the ora serrata to quantify vascular density and reveal the patterns, topography, and key metrics of vascular anatomy. To confine the scope of our analysis, we examined mice aged 13 to 61 weeks, representing the normative vision window^18^ for visual psychophysics and commonly examined ages for disease models such as diabetic retinopathy^4^^,^^6^^,^^7^ and glaucoma.^19^^,^^20^ This placed them beyond the development period that is completed by 2 weeks,^3^^,^^21^ but before noted senescence changes after 52 weeks, such as decreased photoreceptors, dysregulated metabolism,^22^^,^^23^ and decreased branching and vessel length within the intermediate and deep layers.^24^ Although often overlooked, we also analyzed the axial vessels that bridge plexuses as they play an essential role in circulating blood deeper into the retina. With such analyses, we later discuss and associate these densities with the neural populations of the mouse retina that they serve. This establishes a robust baseline of healthy adult mouse retinal circulation during the most relevant period for both vision and disease.

Last, we compared in vivo imaging with our ground-truth ex vivo dataset. We show that single-cell resolution in vivo imaging can accurately measure the same metrics used ex vivo. This enables longitudinal study free of histological artifacts in an intact and perfused network. Together, such metrics and anatomy described in each of the plexuses and their interconnecting vessels serve as a normative biomarker that can be traced and compared to conditions of disease and pathology.

## Methods

### Animals

This study used 18 (15 ex vivo; 3 in vivo) healthy male mice (The Jackson Laboratory stock #008241, Bar Harbor, ME, USA) aged 13 to 61 weeks. Males were chosen to match with an ongoing diabetic study. They were divided into age groups: 11 to 20 weeks, 21 to 30 weeks, 31 to 40 weeks, 41 to 50 weeks, and 50 weeks and older. Mice were housed under diurnal lighting conditions (12-hour light/dark cycle) and fed ad libitum. All guidelines from University Committee on Animal Resources at the University of Rochester, Rochester, New York, and the ARVO statement for the Use of Animals in Ophthalmic and Vision Research were followed.

### Eye Enucleation and Dissection

Mice were sacrificed using CO_2_ inhalation and secondary cervical dislocation. Eyes were immediately removed without perfusion and fixed for 12 to 18 hours in 4% paraformaldehyde. The RPE was separated before four radial cuts were made to create a flat mount. They were placed vitreous side up on slides using an antifade mounting medium (VECTASHIELD, Vector Laboratories Inc., Burlingame, CA, USA).

### Confocal Imaging

Eyes (*n* = 15) were imaged using a confocal microscope (Nikon A1 Ti2 Eclipse). Montages at 20× magnification were used to locate regions of interest and orient their location relative to the optic disc across four zones: 0 to 0.5 mm, 0.5 to 1.0 mm, 1.0 to 1.5 mm, and 1.5 to 2.0 mm. We imaged high-resolution *Z*-stacks at 60× magnification (Plan Apo λ 60x Oil) using 300 µm × 300 µm squares with a 0.1-µm step size. A minimum of 4 *Z*-stacks were collected per retina for a total of 80 square stacks and 1 continuously imaged strip (2500 µm × 300 µm; 0.1-µm step size).

### ImageJ Neuroanatomy SNT^25^ Tracing

A single vessel was defined as a continuous path with no branching; any branch point signaled the end of one path and the start of at least two more. These paths were manually labeled as the superficial, superficial-to-intermediate (SI) region, intermediate, intermediate-to-deep (ID) region, or deep. Seventy-nine of 80 cubes were analyzed in their entirety. One cube was cropped as a result of localized histological distortion. To compensate for varying tissue shrinkage, we normalized depth between two features present in every stack: the bottom of the GCL and the top of the outer nuclear layer (ONL).

### Vascular Metrics

We quantified total vessel length (mm), depth of each layer (µm), layer thickness (µm), vascular density (mm/mm^2^), vessel segment length (µm), and vessel segments per area (number/mm^2^). Each metric was calculated based on *X*, *Y*, and *Z* coordinates exported from SNT traces and custom MATLAB code.

When exported, the coordinates were associated with their manually assigned (superficial, SI region, intermediate, ID region, deep) and if they belonged to the same vessel segment or path. We summed vessel lengths using the distance formula. These sums could be calculated as a function of label or depth. To calculate totals as a function of label, all paths from each label were summed for the total path length. To calculate totals as a function of depth, we normalized *Z* due to known histological consequences that resulted in tissue shrinkage.

Znormalized=(Z-Z∑min)(Zmax-Zmin)

Here, *Z* was the original *Z* coordinate, *Z_min_* was the *Z* coordinate of the top of the ONL, and *Z_max_* was the *Z* coordinate at the bottom of the GCL. All paths with the same *Z*-coordinate were summed. *Z_normalized_* was then scaled by 95 µm based on the reported anatomical distance between the GCL and ONL.^26^ The depth of each vessel layer was determined as the maximum of each aggregated peak using cross-section averaging. The thickness of the vascular layer was measured as the full width at half maximum of each peak in the axial dimension.

We calculated a label-based density and a *Z*-coordinate, label-independent density. This is because unlevel samples and node-to-node vessel segments could appear to belong to multiple layers, and thus neither method was a perfect representation. Therefore, we averaged the two. Vascular density was scaled from the total vessel length per *Z*-stack area (300 µm^2^) to the total vessel length per square millimeter.

Metrics that were vessel specific used the manually labeled data. Vessel segment length was the distance between two branch nodes or a single SNT path. The number of branches was the count of individual SNT paths per area, scaled from the *Z*-stack area to 1 mm^2^. The total length of vessels in the retina was the vessels per square millimeter multiplied by 16 mm^2^, the average area measured from 15 mice (16.17 ± 2.53 mm^2^). This value also had good agreement with past reports.^2^ Qualitative coarse diameter measurements were made using the ImageJ line measuring tool across the largest vessels of the plexuses for descriptive purposes. These measurements were aided by the distinct morphological features visualized using NG2, such as banding on arterioles, stellate patterns on venules, and the protruding somas and processes of pericytes on capillaries.^15^

### Axial Vessel Naming and Classification

We classified the branching patterns of the axial vessels into five fundamental types. SI-connecting vessels linked the superficial and intermediate layers. ID-connecting vessels linked the intermediate and the deep layers. SID-connecting vessels linked superficial, intermediate, and deep layers. SID differed from SI + ID as the vessel linking the superficial to the intermediate had to be less than 2 µm from the vessel linking the intermediate to the deep. SD-connecting vessels directly linked the superficial and deep layers, bypassing the intermediate layer. Interplexus branching vessels could link any of the plexuses, but had to split into two or more branches prior to its destination, often within a nuclear layer.

### Mouse Preparation for In Vivo Imaging

Mice were anesthetized using an intraperitoneal injection of ketamine and xylazine (intraperitoneal: 100 mg/kg ketamine, 10 mg/kg xylazine), and kept anesthetized during imaging by delivering 1% v/v isoflurane with supplemental oxygen through a nose cone. Body temperature was maintained at 37°C via a heat pad. We intraperitoneally injected fluorescein (0.1 ml of 10:1 dilution 10% AK-FLUOR; Akorn, Lake Forest, IL, USA) to visualize the complete vasculature. Pupils were dilated with eye drops of 1% tropicamide (Sandoz, Basel, Switzerland) and 2.5% phenylephrine (Akorn). To maintain eye hydration and provide an optical interface, a +10 diopter rigid contact lens with a base curvature of 1.6 mm (Advanced Vision Technologies, Lakewood, CO, USA) was placed over the cornea. GenTeal (Alcon Laboratories, Inc., Fort Worth, TX, USA) was administered around the contact to sustain the hydration. A stereotactic stage with five degrees of freedom allowed both the centration of the pupil and retinal navigation.

### Adaptive Optics Scanning Light Ophthalmoscope (AOSLO) Imaging

We used a previously described custom-built AOSLO^7^^,^^27^ with simultaneous coplanar phase contrast and fluorescence imaging capabilities. Briefly, it has three light sources: (1) a 904-nm diode source (12 µW, Qphotonics, Ann Harbor, MI, USA) for wavefront sensing; (2) a 796-nm superluminescent diode (214 µW, Superlum, Cork, Ireland) for reflectance imaging in phase contrast mode; and (3) a 488-nm light source (56 µW, Toptica Photonics, Farmington, NY, USA) for fluorescence that were co-axially combined. A 15-kHz fast resonant scanner and a slow 25-Hz galvanometer relay a raster scan on the mouse pupil through a series of five afocal telescope pairs. Optical aberrations induced by the mouse eye were calculated using a Shack Hartman Wavefront Sensor and corrected for via a 97-actuator deformable mirror (ALPAO, Montbonnot-Saint-Martin, France). Simultaneous reflectance and fluorescence detection was achieved with two photomultiplier tubes (H7422-50 [reflectance]; H7422-40 [fluorescence]; Hamamatsu Photonics, Hamamatsu, Japan). For fluorescence, we also used a 25-µm pinhole and a 520Δ35 band-pass filter (FF01-520/35-25, Semrock, Rochester, NY, USA).

Cartesian videos were acquired with a 5.01° × 3.89° field of view for 10 seconds at multiple depths, each 7.3 µm apart, mirroring the protocol used ex vivo. We imaged two locations from each mouse, one within 0.5 mm of the optic disc and the other at 1.0 mm eccentricity. Using a custom registration program, we motion corrected and averaged the videos to create a single image with higher signal-to-noise ratio. These images were combined into a *Z*-stack and traced with ImageJ SNT. Arteries and venules could be distinguished via the flow direction: away or toward the optic disc. Capillaries were vessels less than 7 µm, which are primarily single file flow.^28^

### Statistical Comparisons

All statistics were calculated using GraphPad Prism version 10.1.2 for Windows (GraphPad Software, Boston, MA, USA). We used repeated measures ANOVAs for trilaminar effects, Welch ANOVA for age or eccentricity effects on total vessel density, and mixed-effects models for age or eccentricity effects on the different trilaminar layers. We also performed a single three-way ANOVA to compare trilaminar layer × age × eccentricity. Simple linear regression was used to estimate the magnitude of age effects. All results were considered significant at a *P* value of less than 0.05. All values indicate mean ± 1 SD.

## Results

### Mouse Retinal Vasculature Exhibits a Trilaminar Profile

**Figure 1.**
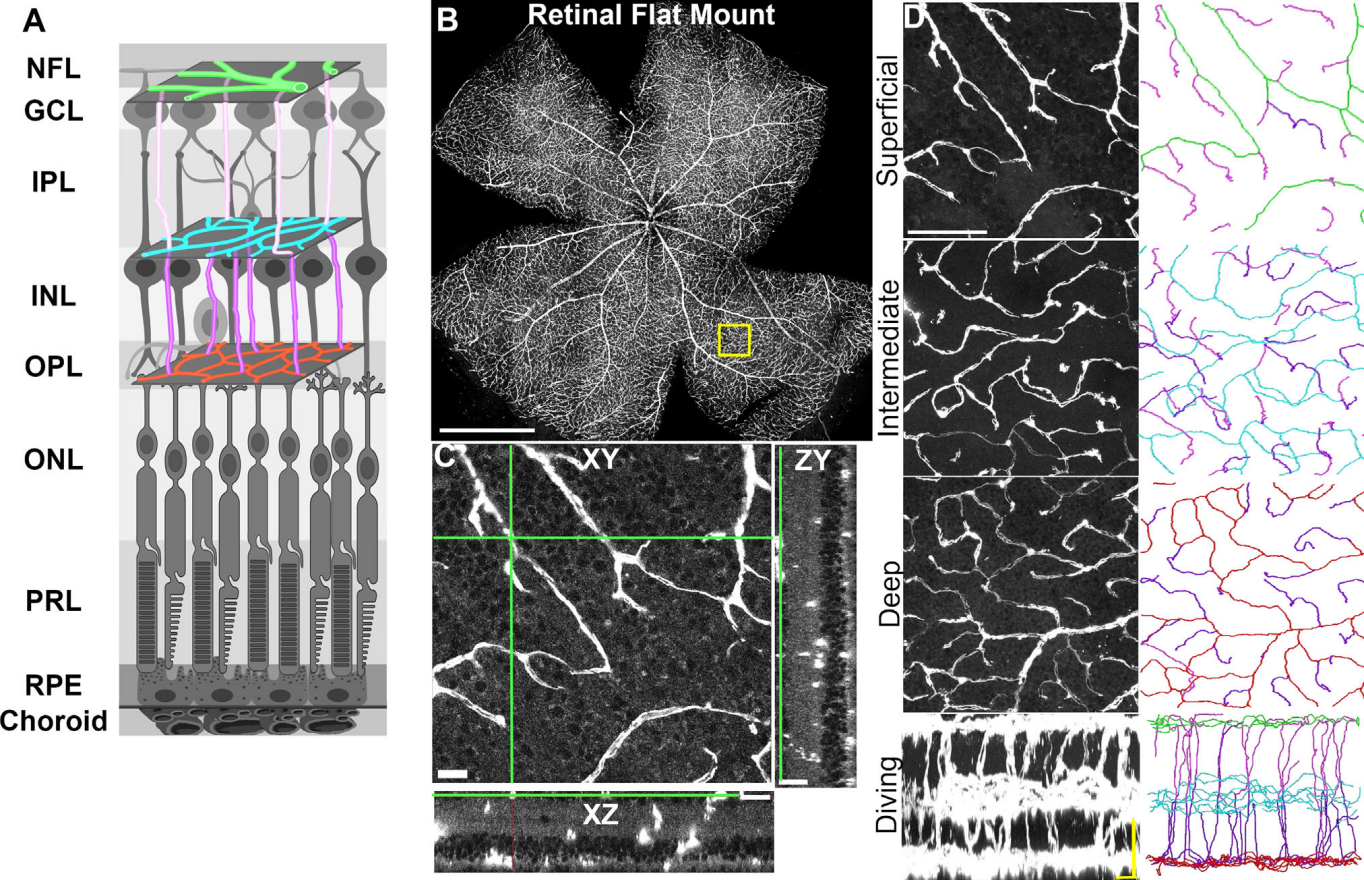
NG2 fluorescent label reveals three clear vascular layers with axial bridges. (**A**) Retinal diagram created with Biorender.com. IPL, inner plexiform layer; INL, inner nuclear layer; NFL, never fiber layer; OPL, outer plexiform layer; PRL, photoreceptor layer. Choroid; superficial plexus (*green*); superficial to intermediate region (SI region; *light pink*); intermediate plexus (cyan); intermediate to deep region (ID region; *purple*); deep plexus (*red*) (**B**) A 20× retinal flat mount montage. Scale bar, 1000 µm. The *yellow box* outlines the region of interest shown in (**C** and **D**). (**C**) *XY*, *ZY*, and *XZ* slices. The *green* crosshair represents the superficial plexus. (**D**) XY images (*left*) showing the SID layers with their corresponding color-coded traces (*right*) exported from the ImageJ plugin SNT. Axial connections were shown in *XZ* view for better visualization. Scale bars, 25 µm.

We imaged 81 locations across 15 mice at different adult ages and eccentricities, and found a clearly organized, highly stable vascular network at all ages and locations (Fig. 1). Three stratifications of vessels largely avoided the high cell density nuclear layers and resided mainly within the plexiform layers (Supplementary Figs. S1 and S2; Supplementary Video S1). The most superficial stratification was in the nerve fiber layer with some interdigitation with the GCL, the intermediate stratification was in the inner plexiform layer, and the deep stratification was in the outer plexiform layer. The intermediate and deep layers closely bordered the top and bottom of the inner nuclear layer. Traversing the nuclear layers, a small fraction of vessels formed axial connections through the GCL and inner nuclear layer. Anatomically, we described these regions as the SI region and ID region, respectively, based on their connections with the layers above and below. And, like in many vascularized retinas,^29^ no vessels protruded into or below the ONL.

Visualization of these stratifications was possible by imaging NG2 DsRed transgenic mice that showed strong, continuous labeling of the vascular tree. Although there have been questions about how far down the vascular tree NG2 is expressed,^30^ we traced all vessels from arteriole to capillary to venule without a loss of traceable fluorescence. Our analysis revealed a continuum of paths with very few terminating into a dead end (<1%), suggesting the label was sufficient to trace the entire vascular tree. This strong fluorescence, combined with high axial and lateral resolution (axial, 649 nm; lateral, 216 nm), ensured that all vessels were measured (Supplementary Figs. S3 and S4; Supplementary Video S2).

### Total Path Length of the Mouse Retinal Circulation

**Figure 2.**
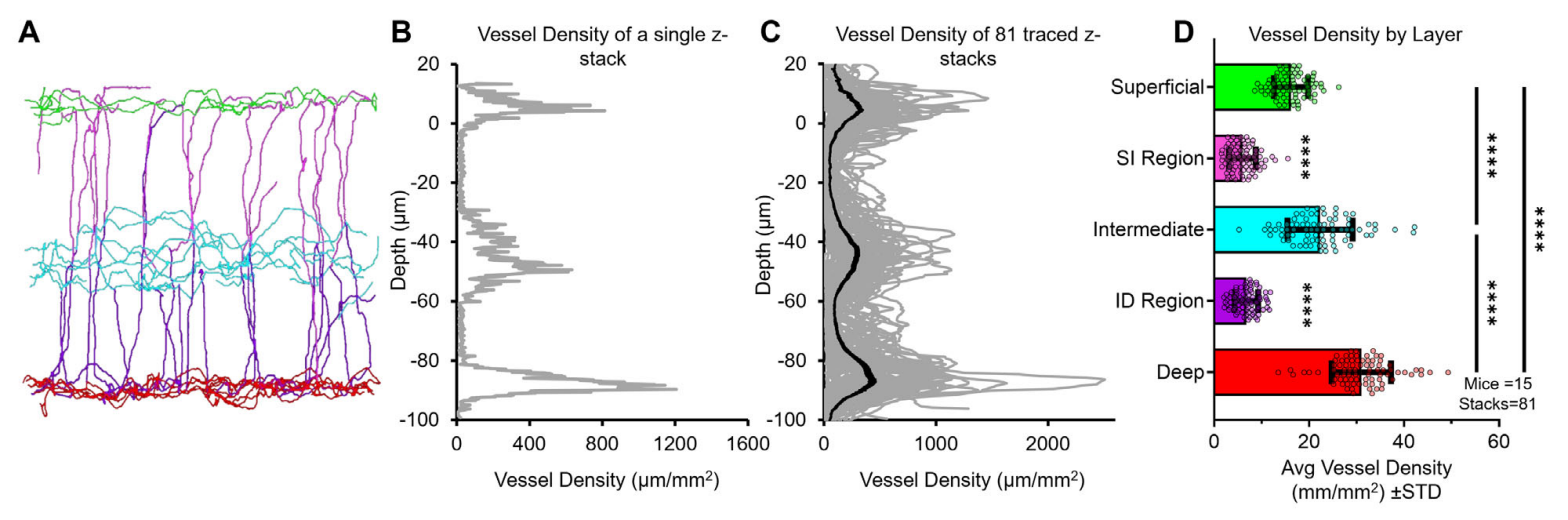
Three high-density peaks represent the superficial, intermediate, and deep layers. (**A**) One *Z*-stack with 188 vessels exported from ImageJ SNT color-coded by label: superficial (*green*), SI region (*pink*), intermediate (*cyan*), ID region (*purple*), and deep (*red*). (**B**) Quantification of the vessel density data shown in (**A**) as a function of depth. (**C**) Quantification of all 81 *Z*-stacks showing consistent prominence of three peaks. Individual stacks in gray, average of 81 locations in black. (**D**) The average vessel density per square millimeter of retinal tissue. The SI and ID regions were significantly different from S, I, and D. ****P < 0.0001.

All 81 locations showed three distinct peaks of high vessel density localized to the same retinal layers regardless of subject, age, or eccentricity (Fig. 2). In total, we calculated the total vascular path length to be 1.29 ± 0.24 m per mouse retina. This total was contained within eyes 4.52 ± 0.38 mm in diameter and covered a total area of 16.17 ± 2.53 mm^2^. Thus, 1.3 m of vessels were tightly organized within this signature trilaminar structure.

### Key Similarities and Differences in the Three Vascular Layers

Trilaminar organization was consistent across all subjects, ages, and locations. All stratifications exhibited lateralized flow, that is, the primary direction of flow was parallel to the en face plane. Each stratification was confined to relatively narrow bands, suggesting a developmental or functional role of their position relative to specific retinal layers. However, there were also notable differences in the structure and vascular density between the layers.

The superficial layer consisted of the largest (>30 µm) arterioles and venules radiating from the optic disc. The arterioles subsequently bifurcated, usually creating a narrow angle branch-like appearance on the arterial side and larger angle intersections on the returning venule side. Near the largest arterioles, we observed fewer microvessels akin to a capillary-free zone described in humans.^31^^–^^33^

Unlike the superficial layer, the intermediate layer consisted almost entirely of microvessels and contained no vessels larger than 20 µm. It had a unique connective network with a high number of lateral and axial connections, that is, the shortest path between adjacent lateral vessels may require an axial vessel. However, the intermediate layer was not simply the junctional zone of axial vessels; like the other layers, it exhibited a dominant en face lateral flow (Supplementary Fig. S3 and S4, side view).

The deep layer was also dominated by a lateralized vessel network. In 81 samples, no vessels larger than 20 µm were found. Visually, its connections were more mesh-like or highly anastomotic; it contained many more lateral loops where traced paths diverged before converging again (Supplementary Fig. S3 and S4, en face view).

Quantification of key retinal parameters is provided in Table. On average, vessel density increased with greater distance from the vitreous, and both the total vessel path length and number of branches per square millimeter increased. However, the length of individual segments decreased with depth. A single unbranched vessel was both a fundamental unit of vessel anatomy and the smallest theoretical unit of neurovascular coupling, whereby blood can be diverted between two vessels and differentially impact downstream regions. The magnitude of this decrease was small, all segments averaging 55 µm, suggesting the retina favored a similar degree of control over a larger number of vessels as depth increased.

**Table.** Characteristics of the Trilaminar Mouse Retinal Vasculature

Metric	Superficial	SI Region	Intermediate	ID Region	Deep	All
Vessel density (mm/mm^2^)	16.14 ± 3.61	5.96 ± 2.83	22.14 ± 6.86	6.77 ± 2.48	31.01 ± 6.24	82.02 ± 15.64
Vessel length (µm)	61.34 ± 17.78	62.95 ± 13.19	53.27 ± 8.84	62.52 ± 13.94	49.56 ± 10.50	57.93 ± 7.68
No. of branches (count/ mm^2^)	244.80 ± 79.76	143.80 ± 61.72	429.50 ± 162.10	165.80 ± 66.15	603.80 ± 231.00	1587.64 ± 477.84
Region thickness (µm)^*^^,^^†^	10.65	34.91	19.08	23.49	14.4	102.53
Normalized depth (µm)^*^	4.41	–	−44.13	–	−86.9	–

* Depth is relative to the bottom of the GCL and measured from the averaged population seen in Figure 2C; no STD reported.

† Full width at half maximum.

Values are mean ± SD.

### Axial Connections Link the Three Predominant Stratifications

Bridging the three layers was a small population of vessels with a predominantly axial organization. Topographically, they spanned between the superficial and intermediate layers (SI region, passing through the GCL) and between the intermediate and deep layers (ID region, passing through the inner nuclear layer). Despite their critical importance, these axial vessels are often overlooked or neglected in both ex vivo histology sections^1^^,^^34^ and in vivo analyses such as optical coherence tomography angiography.^35^^,^^36^ Without them, the arteriole-free intermediate and deep layers could not access this oxygen-rich blood supply. And when quantified, we found that the axial vessel density was 15.36 ± 4.37% of the total density by length. This percentage, while low, was appreciably nonzero and needed further analysis.

**Figure 3.**
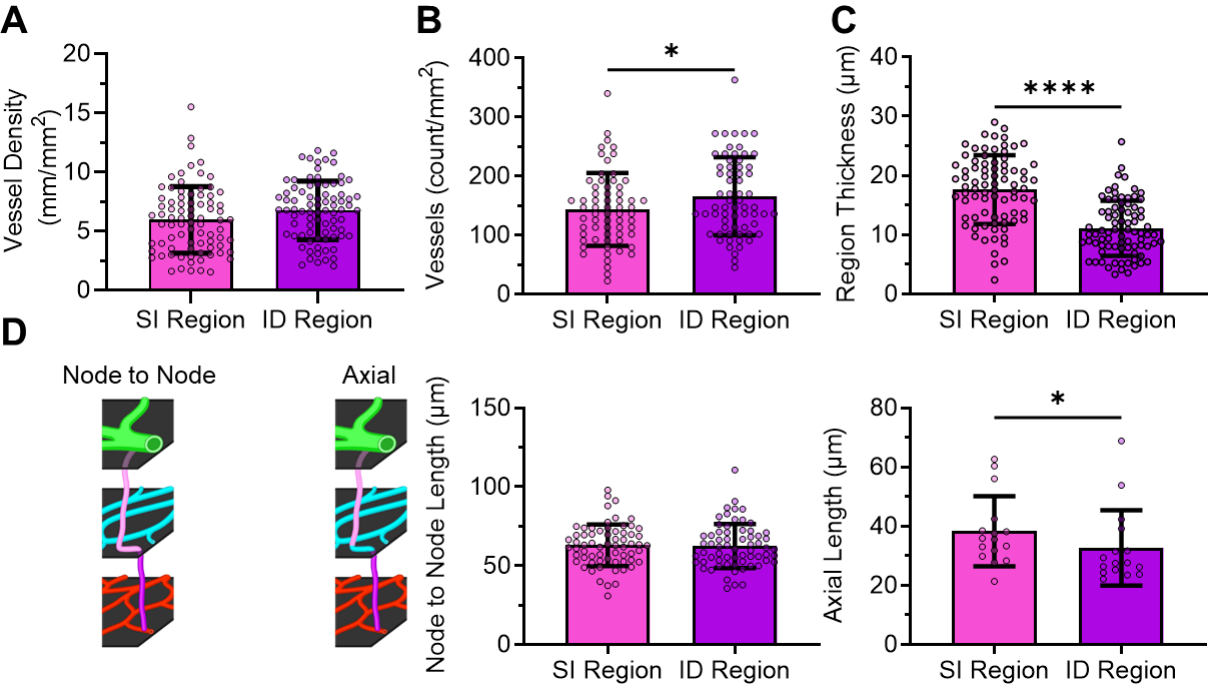
The ID region had a slightly different structure compared with the SI region. The comparison of the SI region and ID region across four metrics: (**A**) vessel density, (**B**) number of vessels per square millimeter (*P < 0.05), (**C**) thickness (****P < 0.0001), and (**D**) vessel length between branch nodes (*middle*) or of the axial component (*right*). A diagram of the length considered (*right*) for the two measurements. (**A–C**) Used all ex vivo data 15 mice and 81 stacks, (**D**) used 15 mice and 65 stacks (*middle*) and 8 mice and 16 stacks (*right*).

The SI and ID regions had similar vessel lengths of 62.73 ± 13.52 µm. However, the arrangement of these vessels differed between the two regions. The ID region had a greater number of branches per area, constrained to a thinner layer (Fig. 3). This indicated that both lateral and axial connections increased in density with depth.

### Axial Vessels Can Be Classified Into Five Anatomically Distinct Types

**Figure 4.**
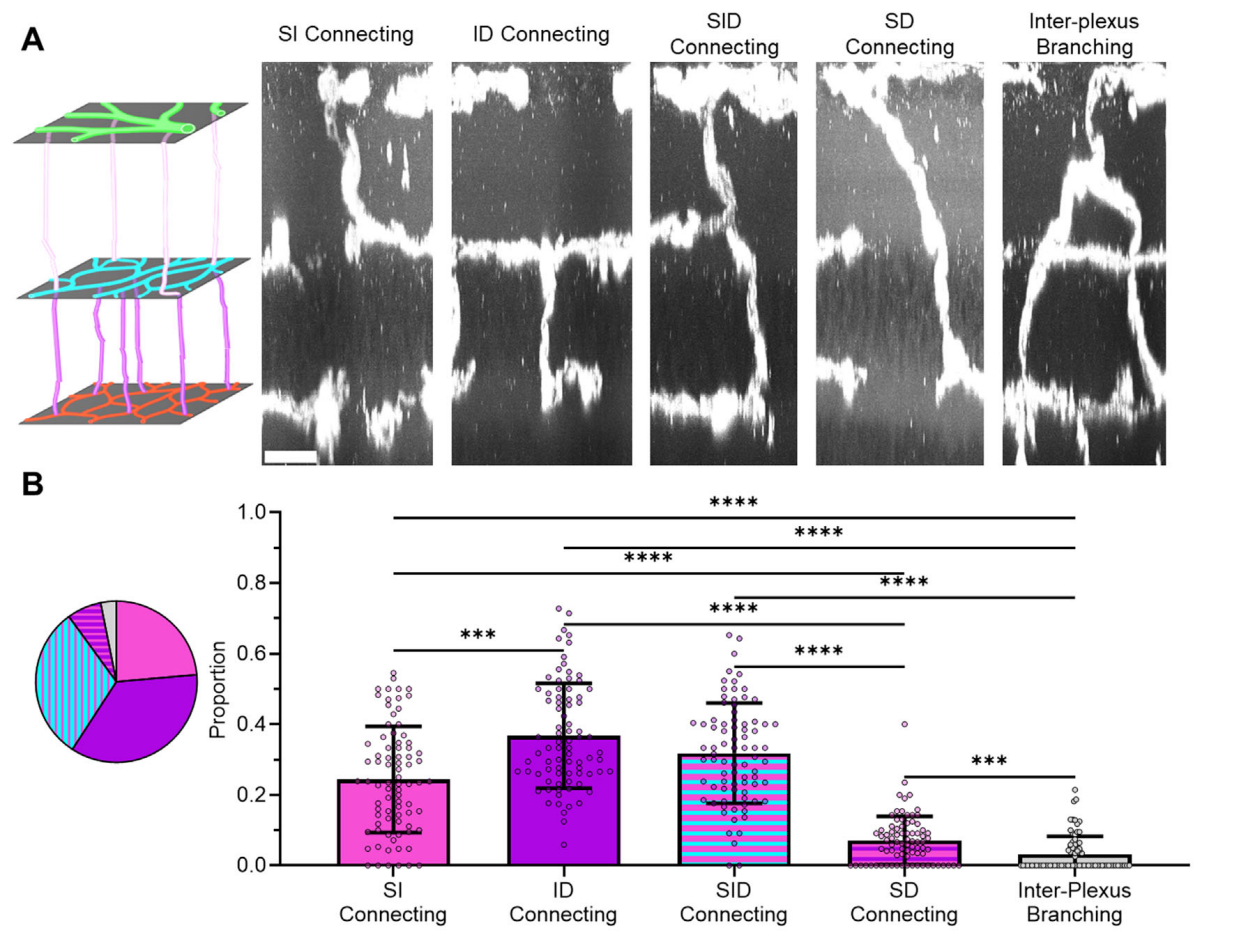
Five different axial connections were represented in different proportions. (**A**) A diagram created with Biorender.com. Superficial (*green*), SI (*pink*), intermediate (*cyan*), ID (*purple*), and deep (*red*) vessels were aligned with example *XZ* sections of each of the five types. Scale bar, 25 µm. (**B**) Pie chart of the five types (*left*) quantified by their relative proportion (*right*). ***P < 0.0004 ****P < 0.0001.

Based on their connective properties, we also identified five anatomically distinct types of axial vessels represented in different proportions (Fig. 4). This semantic taxonomy was logically different and provided different functional potential to shunt and control blood flow between the layers.

1. SI-connecting vessels (24.37 ± 15.05%) were unbranched vessels connecting the superficial to the intermediate layer. Although not quantified, we noted an interesting trend. From arterioles, identified via a bright banding characteristic of vascular smooth muscle cells,^15^ we could see that SI connecting vessels branched early. This finding suggested a direction of flow that dived away from the surface source vessels.
2. ID-connecting vessels (36.72 ± 14.85%) were unbranched vessels connecting the intermediate to deep layers. Without nearby arterioles, it was difficult to discern if these were diving or ascending vessels and were likely a mix of both.
3. SID-connecting vessels (31.80 ± 14.18%) were branched vessels that connected the superficial, intermediate, and deep layers. These differed from a combination of SI and ID connecting vessels based on the amount of lateral traversal in the intermediate layer. To be classified as SID, there could be almost none (<2 µm). This also meant the entire SID connection could be diving or ascending, but not both. In contrast, one SI connecting vessel and one ID connecting vessel could encompass circulation in both directions.
4. SD-connecting vessels (7.11 ± 6.85%) were unbranched vessels that connected the superficial layer to the deep layer and bypassed the intermediate layer. Some of these vessels were linked to venules and were returning to circulation. However, others did not have a discernable flow direction and could represent specialized blood flow that delivered oxygen-rich blood directly to the deepest layer.
5. Interplexus branching vessels (3.24 ± 5.03%) were branched vessels that could connect any of the three layers. However, this branching had to occur between plexuses in either the SI region or ID region, and many imaged locations did not include any vessels of this type. Interplexus branching vessels created a unique shunting location outside the plexuses, but their exact role, if any, is currently unknown.

### The Trilaminar Structure of the Vasculature Was Maintained at All Eccentricities Beyond the Optic Disc

**Figure 5.**
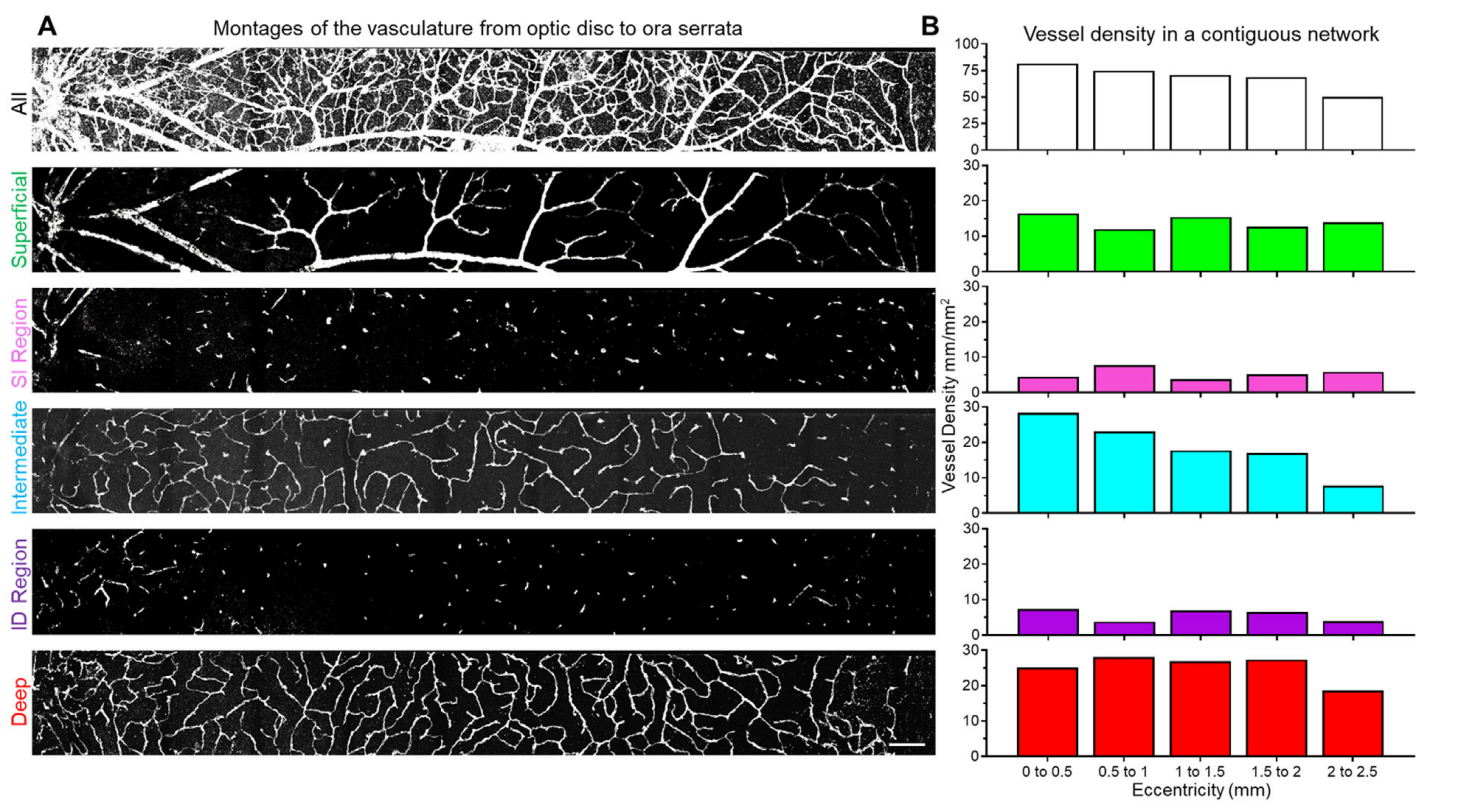
A contiguous vascular network from one retina showed three prominent layers with connecting bridges. (**A**) A 60× image montage of the vessels starting at the optic disc and ending at the ora serrata. All: en face *Z*-projection of the entire stack (*top*) followed by *Z*-projections of each layer. The background was subtracted for visualization purposes. Scale bar, 100 µm. (**B**) Quantification of the vessel densities visualized in (**A**). The total vessel density was calculated in 0.5-mm bins spanning a range from 0 to 2.5 mm.

To better understand a single contiguous network, we examined the vasculature from one retinal flat mount in a mouse that was 58 weeks old (Fig. 5). We quantified the vascular density from the optical axis of the mouse retina where neural density was highest, to the very edge of where photoreceptors may sense light at the ora serrata. We found the trilaminar structure was maintained with high consistency at all eccentricities with no loss or expansion in the number of plexuses.

**Figure 6.**
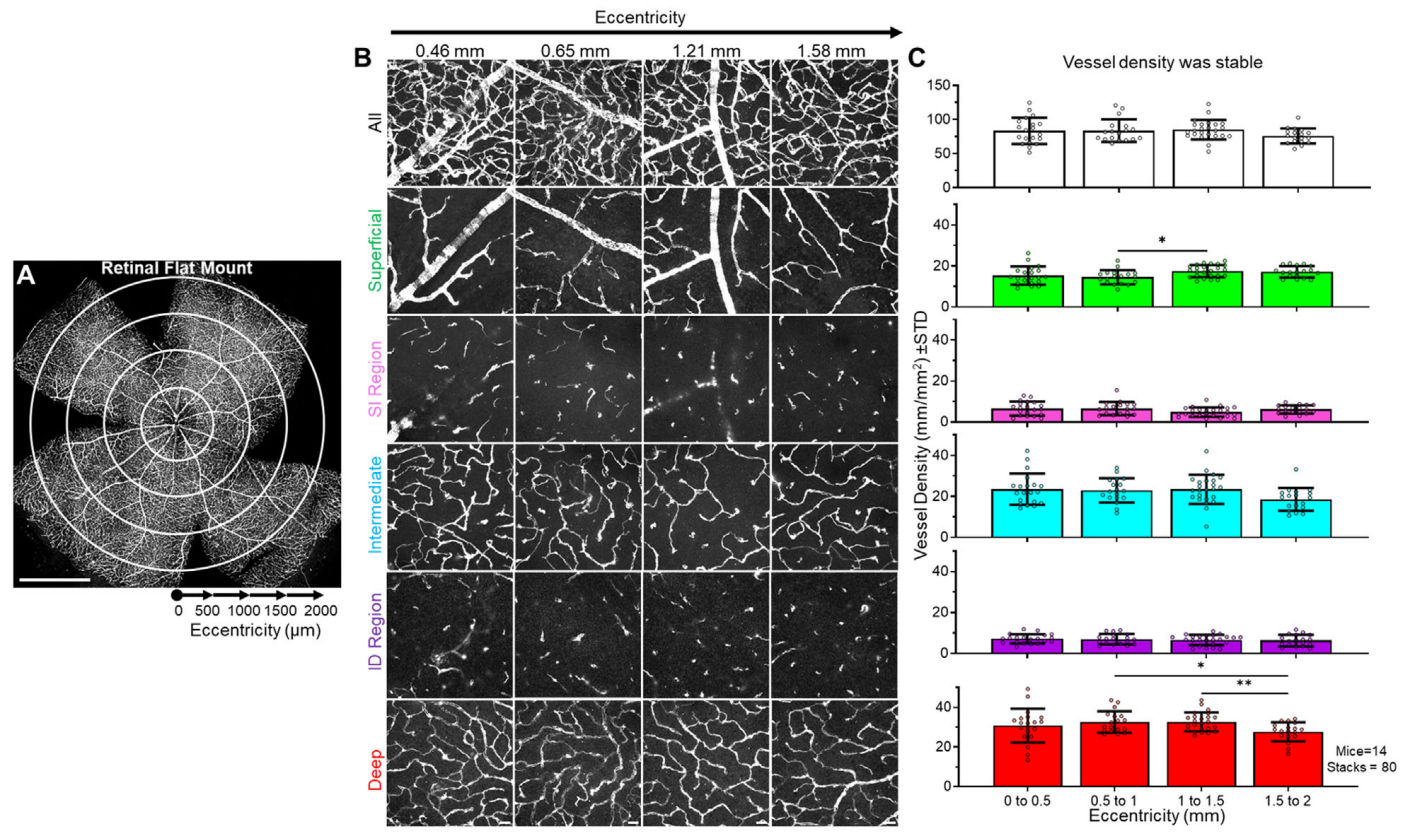
All analyzed mice demonstrated consistent vessel density across the retina. (**A**) Montage of a retinal flat mount with overlayed white concentric showing the four eccentricity zones. (**B**) One region of interest from each bin: 0 to 0.5 mm, 0.5 to 1.0 mm, 1.0 to 1.5 mm, and 1.5 to 2.0 mm was represented as a *Z*-projected of the entire stack (*top*) as well as *Z*-projections of the slices corresponding to each layer. The exact eccentricity of each region of interest was 0.46 mm, 0.65 mm, 1.21 mm, and 1.58 mm, respectively. Scale bar, 25 µm. (**C**) Quantification of the average vessel density. Each circle represented a different stack (mice =14, *Z*-stacks = 80). *P < 0.04; **P < 0.01.

We traced 856 unique paths totaling 52,636 µm of vessel length contained within a 0.75 mm^2^ rectangular regions of interest, yielding a density of 70.37 mm/mm^2^. Extrapolated to the entire retina (16.13 mm^2^), this predicted 1.14 m of total vessels. This total vascular length was divided into the superficial (12.39 mm/mm^2^), SI region (7.78 mm/mm^2^), intermediate (15.84 mm/mm^2^), ID region (8.23 mm/mm^2^), and deep (26.12 mm/mm^2^) layers. These measurements were comparable with the other 80 locations in the remaining 14 mice (Fig. 6; Supplementary Table S1).

Across all 15 mice, we saw no significant effect of eccentricity, F(3,76) = 1.374; *P* = 0.26, but there was an interaction between eccentricity and trilaminar layer, F(12,304) = 3.508; *P* < 0.0001. Tukey's multiple comparisons highlighted only minor changes in the superficial and deep layers (Fig. 6C). We postulated that mouse-to-mouse differences may drive minor changes as we did not observe any consistent increase or decrease in density either within or between vascular layers (Supplementary Fig. S5).

### The Relationship Between Vascular and Neural Density

Unlike in the primate,^33^^,^^37^ the neural density of the mouse is far flatter and less geographically specialized for areas of higher visual acuity. This finding mirrored the consistent vascular density and demarcated stratifications we found and may be a fundamental rule, potentially a direct relationship between neighboring vessels and neural cell types. To examine this relationship and estimate operational principals, we compared the total neural cell density based on numbers from Jeon, Strettoi, and Masland (1998) with the total vascular density.

**Figure 7.**
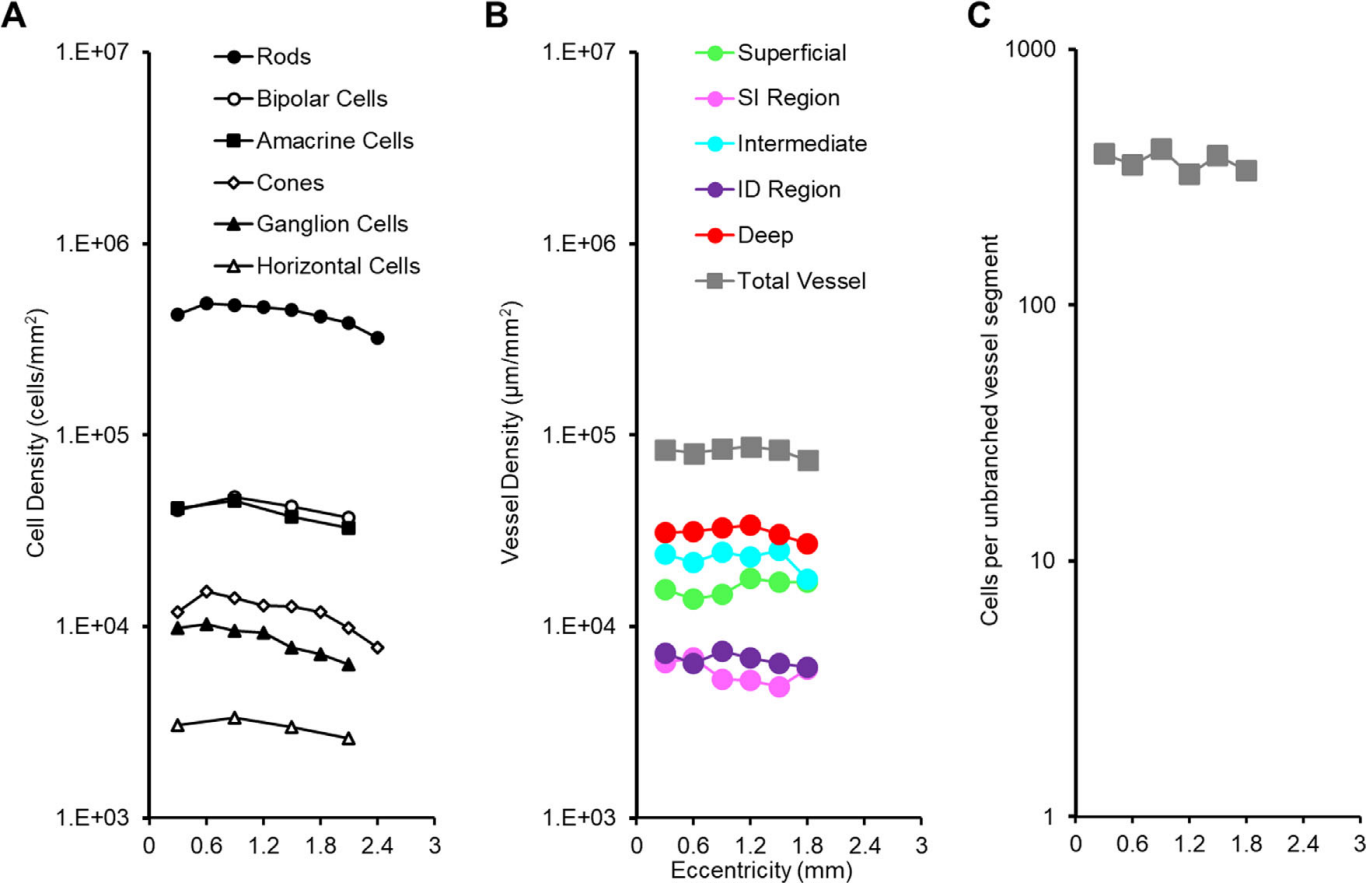
Both neuron and vessel density show a flat and consistent relationship at all eccentricities. (**A**) Adapted recreation of the comparative distributions of the retinal cells figure from Jeon, Strettoi, and Masland (1998) (with author and journal permissions; Copyright 1998 Society for Neuroscience). (**B**) Vessel density for the total and each layer plotted in matching eccentricity bins to (**A**). (**C**) Ratio of the cells to vessel based on the average length of an unbranched vessel.

Their work showed approximately 527,800 total neural cells per mm^2^. Combined with our data, this estimated an average of 367.29 ± 33.30 cells per single unbranched vessel segment (Fig. 7). This ratio sets an estimate of the smallest theoretical unit of neurovascular coupling predicated on two assumptions. One, a single vessel segment cannot modulate blood flow between branch nodes beyond a global constriction or dilation. Two, the interconnections between neural cell types exhibit a primarily axial organization. That is to say, photon detection signals from photoreceptors are relayed in an overall columnar organization. With these assumptions, we estimated that 370 neural cells could be impacted by a single neurovascular event diverting blood at a branchpoint. Although oversimplified, this assumption forms a boundary condition in which more complex models may be developed.

In the assumptions above, the 370 cells were 1 retinotopic unit and did not consider the differential contributions of the various neuron types. To address this issue, we also analyzed the ratios of the different neural cells to the closest vascular stratification, summarized in Supplementary Table S2 and Figure S6. The exact numbers of each cell type per vessel differed. However, we found a similar pattern for the relative proportion of each cell type. Thus, it was likely the 370 cells could be broken down into functional units comprised of a ratio of neuron types.

### Vascular Density Was Maintained Across Early Adulthood

Given that mice are often used to characterize vascular changes in chronic and acute conditions of disease, we also characterized the stability of the healthy trilaminar network over time. Relatively little attention has been given to this epoch of stable adult vision,^18^^,^^38^ instead focusing mainly on development^14^^,^^39^^–^^41^ and late adulthood and senescence.^22^^,^^24^

**Figure 8.**
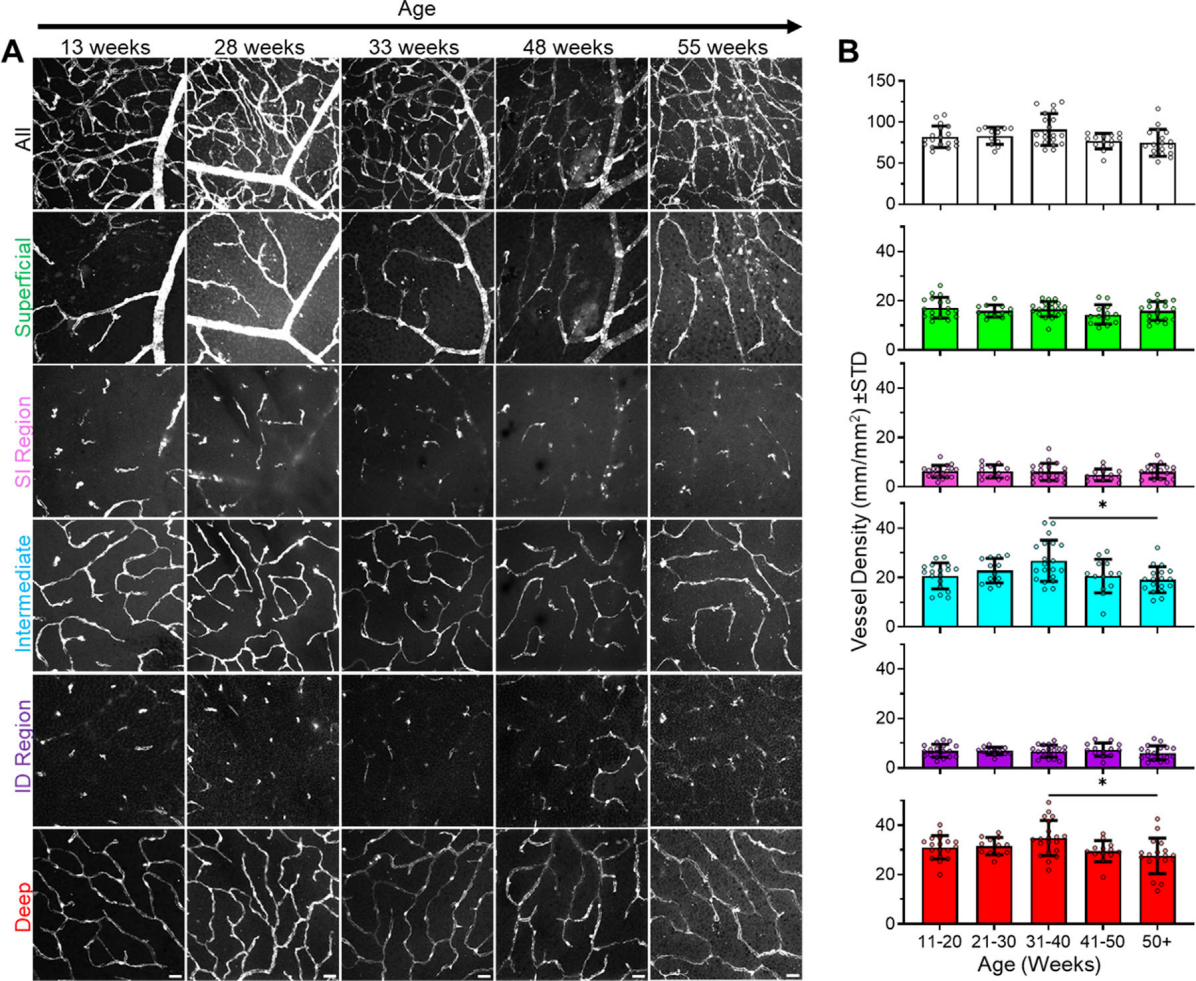
Age did not have a strong effect on vessel density. (**A**) Displayed regions were eccentricity matched and compared across the five different time bins in five different mice. The exact ages shown were: 13 weeks, 28 weeks, 33 weeks, 48 weeks, and 55 weeks. The top row was a *Z*-stack projection of the entire stack, while the rows under it represented each of the vascular layers. 60× magnification; scale bar, 25 µm. (**B**) Average vessel density. Analysis included both the 80 *Z*-stacks and the montaged strip (*n* = 81 stacks, *n* = 15 mice). *P < 0.05.

We found that the retinal vasculature was stable and showed only slight density decreases, most prominent at the latest ages examined, W (4,37.1) = 2.691; *P* = 0.046. However, the magnitude of these changes was small and not noticeable until mice were older than 50 weeks, with a 7.18% decrease in the intermediate and a 6.44% decrease in the deep (Fig. 8B).

### Modeling the Rate of Loss Over Time

To estimate this very low rate of loss, we fit the data with a simple linear regression and found it to be 36 µm/mm^2^ (0.04%) per week. From this rate we could calculate a prediction of effects using a starting point of 40 weeks, which is equivalent to middle age in a human,^42^ and an average retinal area of 16 mm^2^. Using the previously reported metrics (Table), we can estimate a length total of 1.3 m across 25,000 vessels. Assuming 36 µm is lost from every square millimeter, this would total 576 µm or approximately 10 vessel segments. If we combine it with the estimated 370 cells per vessel ratio, it would take 24 weeks, or until 64 weeks of age, to affect 1% of the neural cells. This finding aligns with our data, which reported negligible total vessel density loss within 1 year.

With high stability across both age and eccentricity, we returned to the idea that variation was driven more by individual differences (Supplementary Fig. S7). Using two-way and three-way ANOVAs, we found individual mouse differences accounted for 5.0% variation, age 0.5%, and eccentricity 1.0%. The full list of statical comparisons can be found in Supplementary Tables S3, S4, and S5.

### AOSLO Provides Accurate Estimations of Vascular Density Compared With Ex Vivo Ground-truth

Last, we examined these metrics non-invasively in the living eye. Such imaging enables the potential for longitudinal study free of any histological artifact or tissue shrinkage, and with intact perfusion. Recent advances in ophthalmoscopy provide sub-micrometer lateral and near 10 µm axial resolution^27^ and allows for detailed analysis of the retinal vasculature^15^ (Supplementary Video S3).

**Figure 9.**
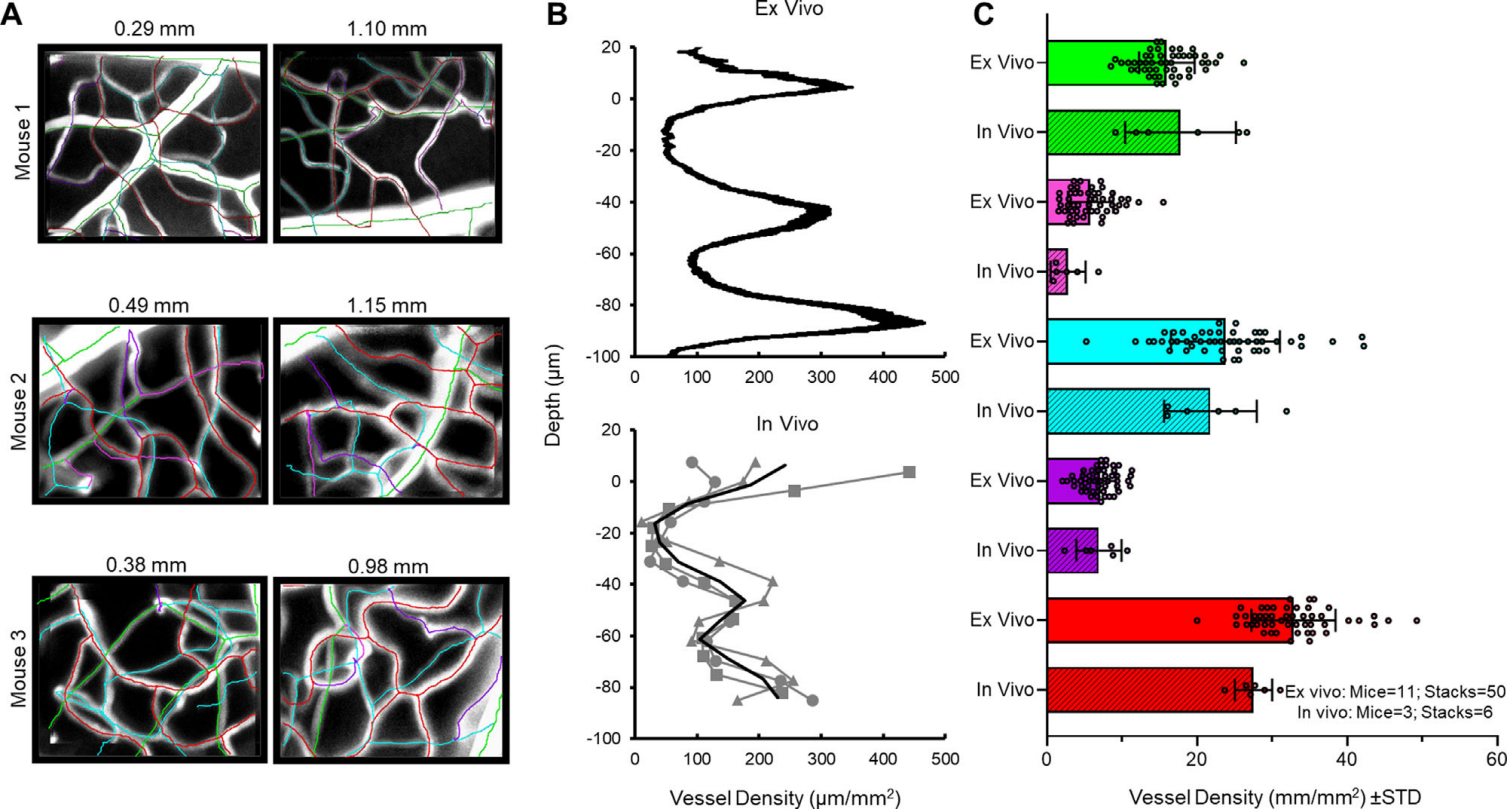
In vivo imaging can obtain accurate measurements comparable to ground truth. (**A**) *Z*-projections of the six locations overlayed with the corresponding ImageJ SNT trace. Scale bar, 25 µm. (**B**) Comparison of the average vessel density by depth between all 81 ex vivo traces (*top*) and the in vivo traces (*bottom*). In vivo mouse averages: 1 (*gray square*), 2 (*gray circle*), 3 (*gray triangle*), and group average (*black line*). 0 on the y-axis represents the bottom of the ganglion cell somas. (**C**) Comparison of the average vessel density by layer between in vivo and ex vivo.

To determine whether accurate in vivo measurements of vascular density and organization could be achieved, we imaged three mice at two locations per mouse. Using fluorescein AOSLO imaging, we saw the three plexuses could be segregated. After tracing and analysis, we found a density profile similar to age and eccentricity-matched ex vivo measurements. Each of the layers had a density of 17.80 ± 7.38 mm/mm^2^ (superficial), 2.82 ± 2.31 mm/mm^2^ (SI axial), 21.79 ± 6.17 mm/mm^2^ (intermediate), 6.93 ± 3.01 µm/mm^2^ (ID axial), and 27.52 ± 2.49 mm/mm^2^ (deep) (Fig. 9). We found that the node-to-node length and number of branches also aligned with ex vivo measurements (Supplementary Fig. S8).

Thus, in vivo measurements of vessel density and length are an exciting opportunity to track vessel perfusion indices over time and in the same animal while avoiding population variance.

## Discussion

We characterized the healthy mouse retinal vasculature during adulthood to provide a comprehensive ground-truth of mouse retinal circulation. This also aided in understanding fundamental rules on how to build, maintain, and optimize the retinal circulation to meet neural demands. Across age and eccentricity, we found the rules governing vessel density, stratification, and axial vessel distribution to be stable overall.

In evaluating this stability, we found that the retinal vasculature favored a specific pattern of organization. First, vessels generally avoid the nuclear layers and predominantly form three stratifications in the plexiform layers, as noted before in mice by Paques et al.^13^ and other species.^37^^,^^43^^,^^44^ Second, the number of branches and vessel density increased with depth. Although speculative, this metric could match with O_2_ measurements taken in many holangiotic mammals that showed a decreased oxygen content with depth.^11^^,^^45^^–^^47^ In this case, increased vessel density increases vessel surface area and coverage to reach more neurons in the deeper retina. Due to its high stability, the vasculature may maintain the density required for the highest concentration of cells throughout the entire retina as a safety mechanism to avoid an oxygen deficit. The importance of such stability was further supported by the limited effect of aging, with our estimates approximating a 2% loss within 1 year. This rate is unlikely to increase greatly, because neural changes have been tracked until 105 weeks with no indication of a sudden acceleration in decline.^22^

From this stability, we speculated there was precise matching of vessels to nearby cell populations. For instance, the superficial layer is located near the RGCs and all axons projecting to the optic nerve, and its relatively low density may maximize oxygen delivery while minimizing vessel intrusion. Meanwhile, the more axially dispersed intermediate layer might reflect subpopulations of amacrine and bipolar cells whose dendritic arbors mainly lie in the inner plexiform layer.^48^ Last, the deep layer interfaces with photoreceptor synaptic output and lies adjacent to their somas. Despite choroid meeting most of the photoreceptors’ demands, the retinal circulation may contribute up to 10%.^45^ Beyond neural cell densities,^2^^,^^22^ Müller glia^49^ and astrocytes^50^ may also drive the vasculature, especially astrocytes that are known to guide its radial outgrowth during development.^40^

Recent work has shown that a unique, previously unclassified RGC subpopulation influences the axial behavior of SI region vessels.^51^ To our knowledge, we quantified all axial vessels for the first time. We noted that the ID region may have a stronger lateral component, perhaps because there is no specialized population driving a more column-like structure. We also identified five types of axial vessels and were intrigued by the possibility that different branching patterns served different roles independent of diving angle. For example, increased branching, especially the rare interplexus branching type, could lead to increased pericytes and allow greater control of blood flow. Greater branching likewise distinguished SI and ID connecting from SID connecting. In contrast, we observed SD connecting vessels with no additional branching and shortcuts superficial to deep or deep to superficial flow. Unquantified intermediate loops may also serve a unique role, because there was axial flow but no movement between plexuses. Beyond the scope of this paper, there remain questions of specific functional differences driven by nearby labeled cell populations or protein differences on the different vessels themselves.

Even without such specificity, the importance of studying the axial components was clear. First, as a consequence of their relative scarcity, we predicted that any axial vessel loss would have a greater impact. It can be estimated by assuming all neurons in the cell-to-vessel ratio would be impacted by the vessel loss. Therefore, losing 1 SI region vessel would affect 2.7 times as many cells as losing one superficial vessel and 4.8 times as many cells as losing 1 intermediate vessel. The loss of 1 ID region vessel had a 3.7 and 5.3 times greater impact than a loss in the intermediate or deep layers. Second, the plexuses have some axial breadth beyond the direct axial connections. We noted that the intermediate layer had a greater thickness than the other two layers and the most axial connections. Therefore, we examined the density in terms of millimeters per cubic millimeter and found that, although the overall pattern of increasing density with depth remained, the intermediate layer had a slightly lower relative density. Additionally, vessel density increased between the SI and ID regions. We saw both effects in our AOSLO imaging data, likely highlighted by the significantly larger *Z*-step size of 7.3 µm compared to 0.1 µm ex vivo.

AOSLO imaging provides the necessary groundwork for in vivo translational data, but mice and human retinas are not identical. Mice have a consistent trilaminar profile, but the number of layers in a human retina varies from one to four with respect to the macula,^52^ which the mouse lacks entirely. Humans may also have compensatory mechanisms in response to age, as increased, not decreased vascular density has been reported in the superficial layer.^32^^,^^33^ This finding highlights the necessity of having well-characterized mouse data first. Fortunately, the high coherence between in vivo and ex vivo data means clinically available instruments like optical coherence tomography^53^ and optical coherence tomography angiography,^35^^,^^54^^,^^55^ used in both humans and mice, can measure the same parameters. Such imaging modalities excel in measuring global blood flow, but struggle with finely sampling the neglected axial dimension. Here, other imaging systems such as an AOSLO or similar adaptive optics usages such as adaptive optics optical coherence tomography^56^ are required to provide the needed resolution. For the first time, we leverage this single-cell resolution to measure vascular metrics in vivo. This resolution provides additional benefit because it can identify arterioles, venules, and capillaries based on the directionality of flow and passage of single-file blood cells in both animals and humans. For future work, the intermediate layer is of greatest interest. It interfaces with many axial vessels and could be a key location in understanding the multiple metabolic regions recently reported in the inner retina.^46^

With this work, we emphasize the importance of examining all three layers and giving additional consideration to the axial connections that have remained largely neglected and possibly uniquely vulnerable. We found that the mouse retinal vasculature is highly stable under healthy conditions and thus a useful biomarker. And its precise ratios with neurons open new avenues to explore their structural coupling.

## Supplementary Material

Supplement 1

Supplement 2

Supplement 3

Supplement 4

## Supplementary Material

**Supplementary Video S1.** Ex vivo image stack. Video shows the fluorescence seen ex vivo starting with the superficial and to the deep. Interdigitating vessels and cell somas are visible. Contrast boosted for visualization purposes. Scale bar, 25 µm.

**Supplementary Video S2.** Three-dimensional vascular cube. Video shows an example of an ex vivo *Z*-stack rendered in 3D and rotated about the y-axis to visualize the three layers and axial connections.

**Supplementary Video S3.** In vivo imaging. Video shows the NIR channel (*left*) and fluorescent channel (*right*) of the same location. Individual blood cells can be seen in both channels. Scale bar, 10 µm.
