# Supplementary material for: Characterization of the Retinal Circulation of the Mouse
Source: Invest Ophthalmol Vis Sci. 2024 Dec 2;65(14):3. doi: 10.1167/iovs.65.14.3 (PMC11613998; doi:10.1167/iovs.65.14.3)
Supplement: Supplement 1 [file iovs-65-14-3_s001.pdf]

Supplementary Figures

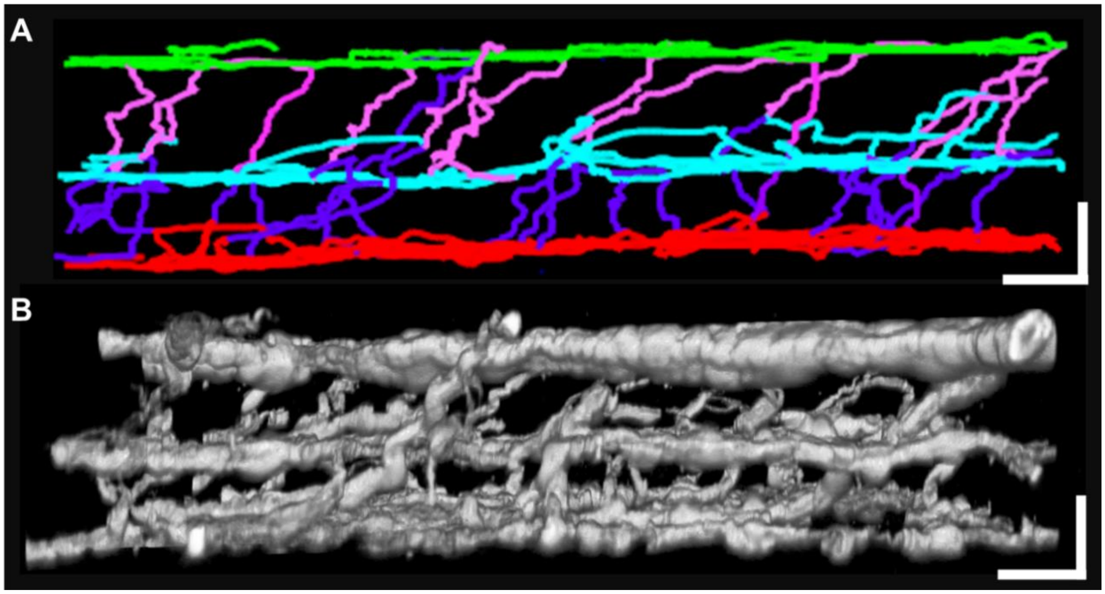

Supplementary Figure S1. Vasculature and corresponding trace

A) Exported trace from ImageJ SNT shown in side view (XZ). **B)** Orientation matched ImageJ 3D viewer of the same Z-stack shown in A. Scale bar = 25 μm

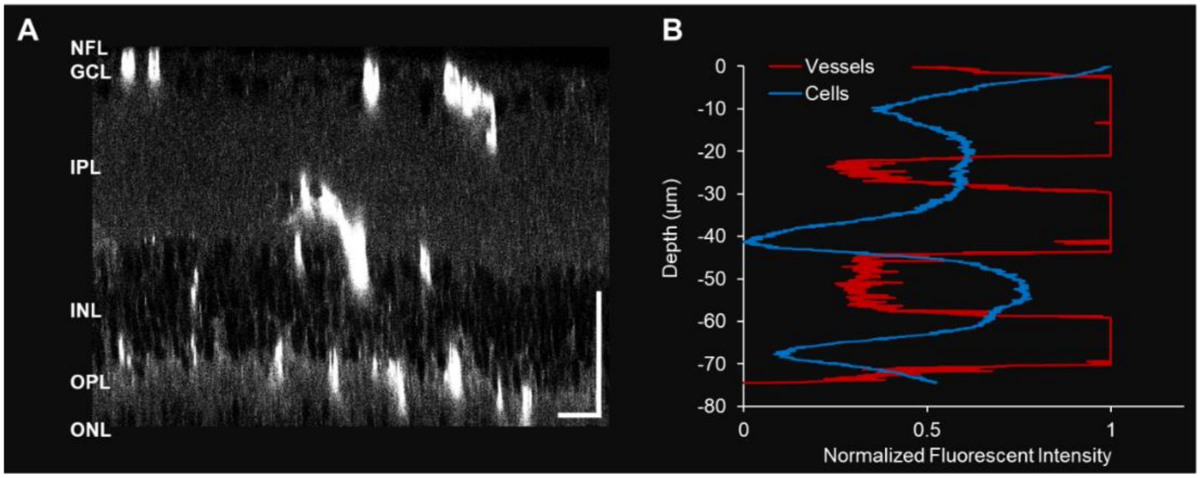

Supplementary Figure S2. Vascular layers avoid nuclear layers

A) XZ view; vessels can be seen in bright white and nuclear soma as black dots. Scale bars = 25 µm **B)** Normalized fluorescent intensity as a function of depth. Depth was the distance from the top of the image stack, which started in the NFL. For visualization purposes, the cell fluorescent intensity was quantified using an inverted image, so the nuclear somas would appear white. The vessels were brighter, so normalization flattened the peak to one.

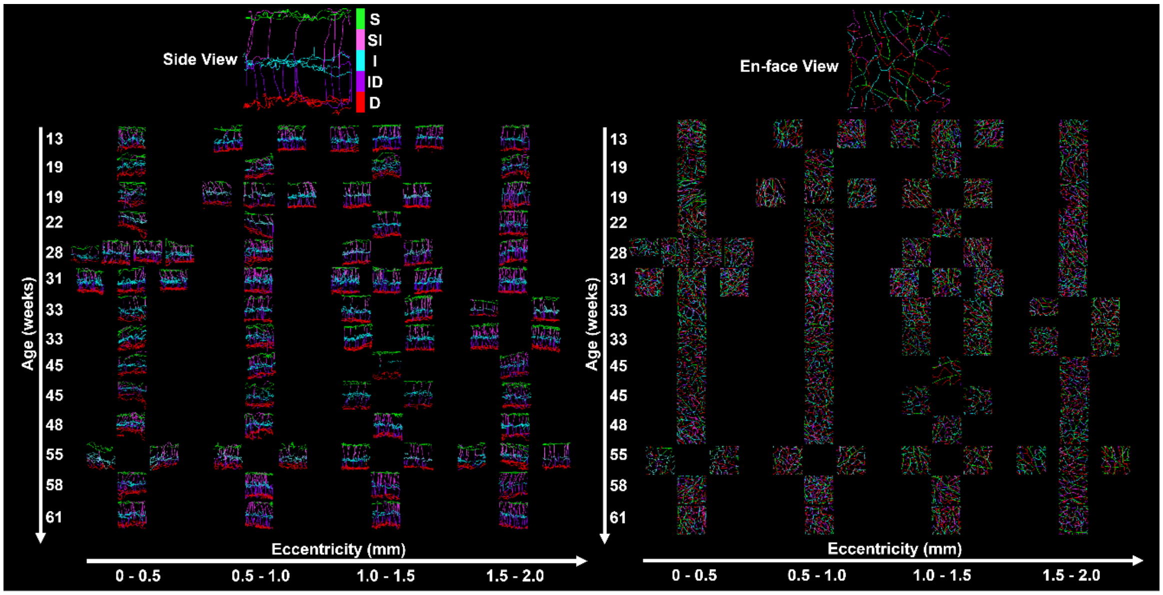

Supplementary Figure S3. All analyzed ex vivo Z-stack cubes are similar when visualized by semantic labeling

Exported traces (n = 80) from ImageJ SNT were arranged to represent the age and eccentricities analyzed. Layers were color-coded: superficial (green), SI Region (pink), intermediate (cyan), ID Region (purple), deep (red). Left: Side view and Right: En-face view. Each row represented a different mouse, and age increased down the rows. Eccentricity increased across the columns from left to right. The four groupings represented the eccentricity groups: 0 to 0.5 mm, 0.5 to 1.0 mm, 1.0 to 1.5 mm, 1.5 to 2.0 mm. Some mice had multiple ROIs within a bracket, which were also arranged in increasing eccentricity.

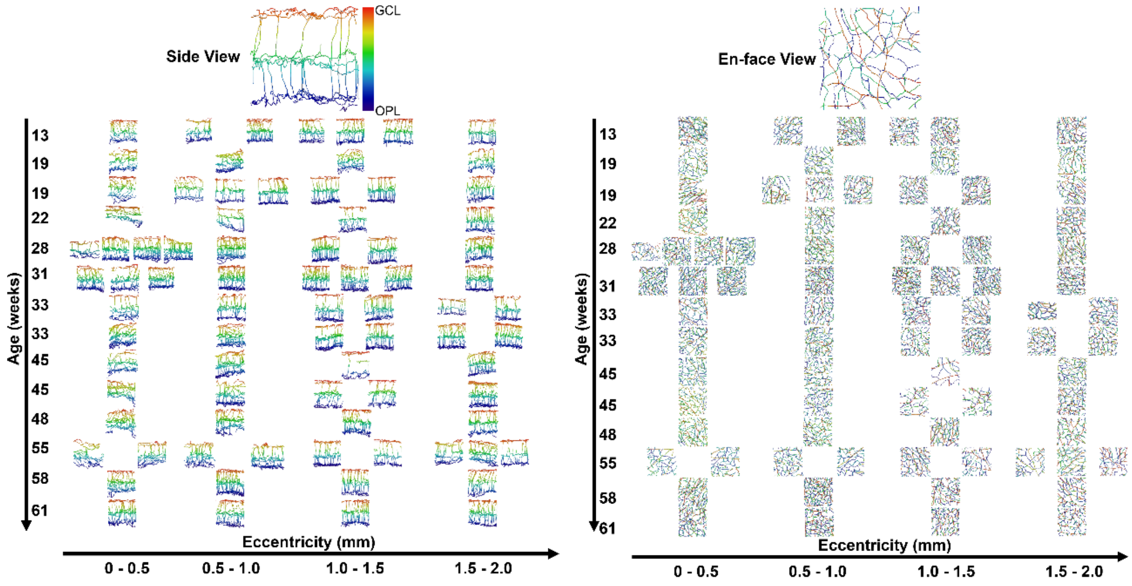

Supplementary Figure S4. All analyzed ex vivo Z-stack cubes are similar when visualized by depth relative to the GCL

Exported traces (n = 80) from ImageJ SNT were arranged to represent the age and eccentricities analyzed. Color-coded (red to violet) by increasing depth from the GCL. Arrangement was identical to Fig. S3. Left: Side view and Right: En-face view. Each row represented a different mouse, and age increased down the rows. Eccentricity increased across the columns from left to right. The four groupings represented the eccentricity groups: 0 to 0.5 mm, 0.5 to 1.0 mm, 1.0 to 1.5 mm, 1.5 to 2.0 mm. Some mice had multiple ROIs within a bracket, which were also arranged in increasing eccentricity.

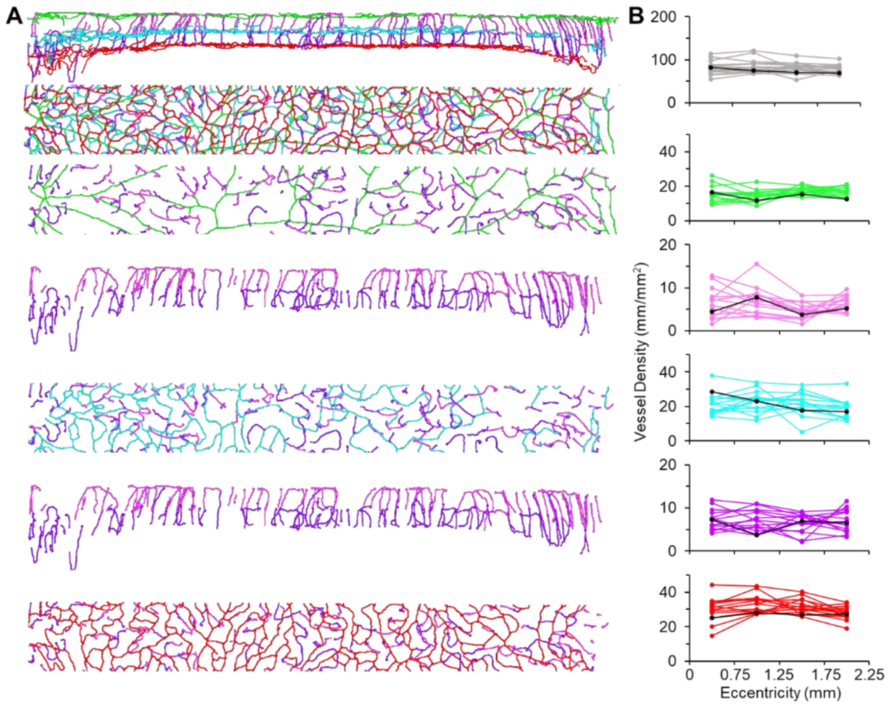

Supplementary Figure S5. There were no consistent eccentricity-driven vessel density changes.

A) Exported traces from ImageJ SNT color-coded by vascular layer. Two merges showed all vascular layers in side view or en-face view. The superficial, intermediate, and deep were represented in en-face views along with axial connections. Axial connections were also shown in side view. **B)** Quantification of the vessel density. Each line was a different mouse with plots colored coded: all vessels (gray), superficial (green), SI region (pink), intermediate (cyan), ID region (purple), and deep (red). The black line represented the data from the contiguous network in A. **C)** Plots of the average (dark) and standard deviation (light) for each of the measurements in B.

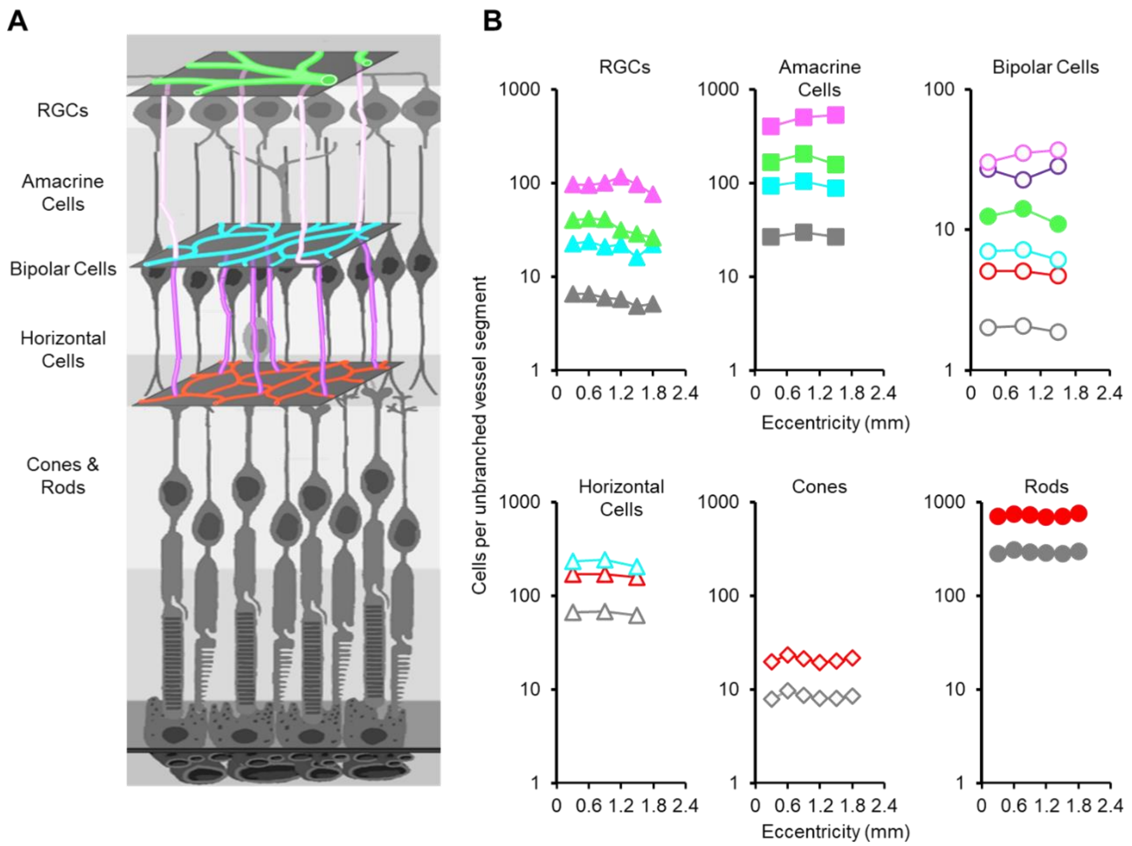

Supplementary Figure S6. The ratios of individual cell types to the nearby vessels across the retina.

A) A representation of the color-coded vessels overlayed on the retinal layers. The major neural cells were retinal ganglion cells (RGCs), amacrine cells, bipolar cells, Horizontal cells, Rods, and Cones. Panel created with Biorender.com. **B)** The ratios of each cell type in A to the nearest vessel types. This ratio was based on the average unbranched vessel length of the superficial (green), SI region (pink), intermediate (cyan), ID region (purple), deep (red), or total (gray). Cell counts were based on numbers reported in Jeon, Strettoi, and Masland (1998).

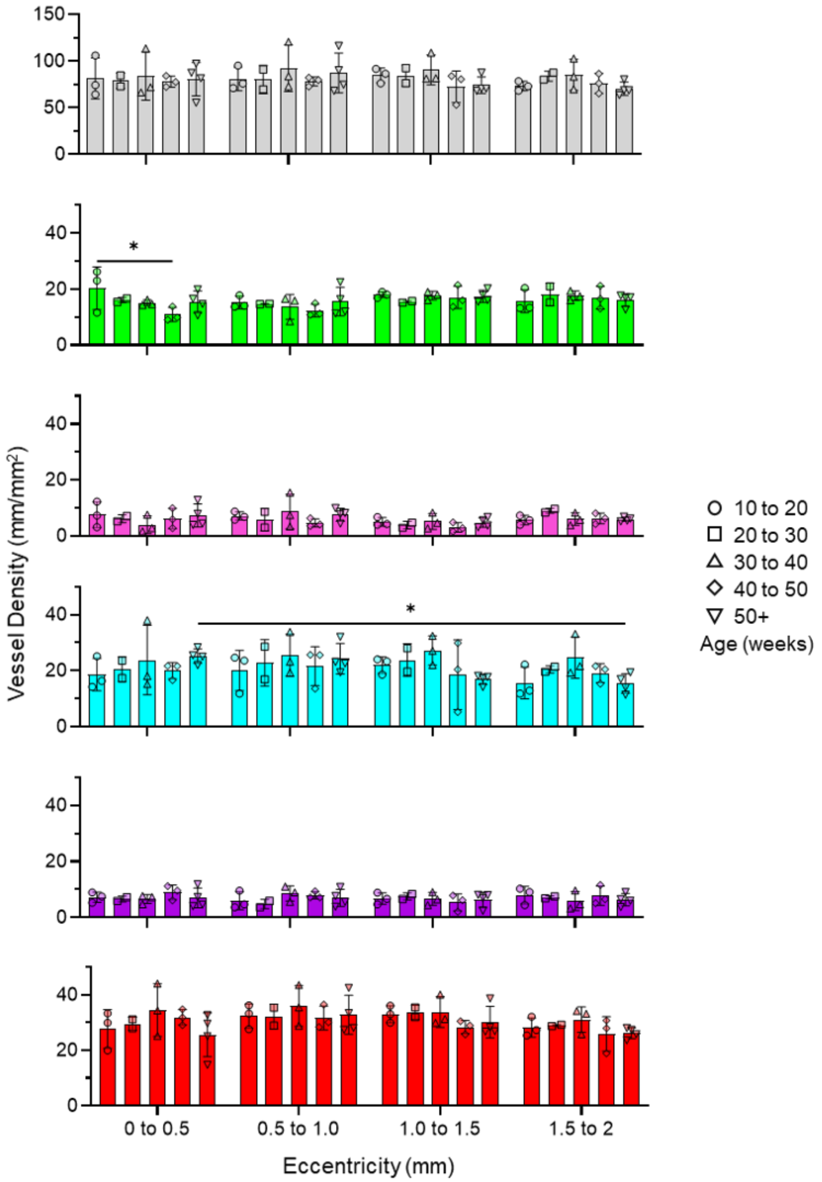

Supplementary Figure S7. There were no strong age or eccentricity effects.

Vessel density of all (gray), superficial (green), SI region (pink), intermediate (cyan), ID region (purple), and deep (red). Each cluster increases in eccentricity from left to right, and within each cluster age increases from left to right.

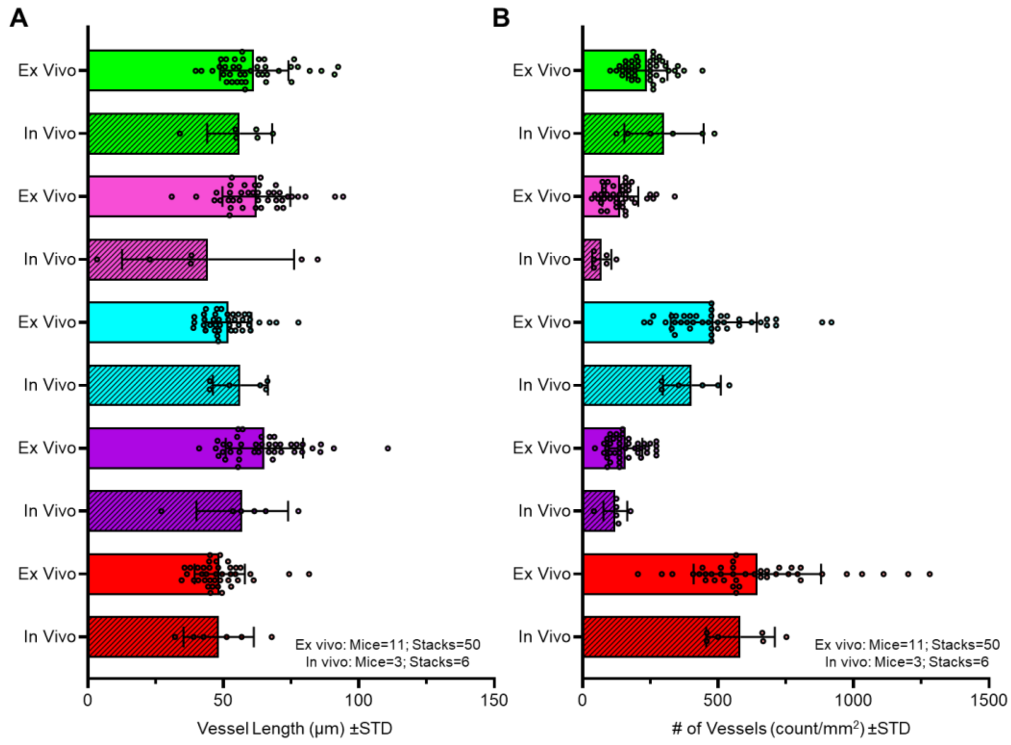

Supplementary Figure S8. In vivo data matches ex vivo ground-truth for vessel length and number of branches

A) Comparison of the average vessel length by layer between in vivo and ex vivo. **B)** Comparison of the average number of vessels by layer between in vivo and ex vivo.

Supplementary Videos

Supplementary Video S1. Ex Vivo Image Stack. Video shows the fluorescence seen ex vivo starting with the superficial and to the deep. Interdigitating vessels and cell somas are visible. Contrast boosted for visualization purposes. Scale bar = 25 μm

Supplementary Video S2. 3D Vascular Cube. Video shows an example of an ex vivo Z-stack rendered in 3D and rotated about the y-axis to visualize the three layers and axial connections.

Supplementary Video S3. In vivo imaging. Video shows the NIR channel (left) and fluorescent channel (right) of the same location. Individual blood cells can be seen in both channels. Scale bar = 10 μm.

Supplementary Tables

Supplementary Table S1. Z-scores of the contiguous network vessel density

Category	Eccentricity	Value	Sample Mean	Z-Score
Superficial	0 to 0.5	16.46	15.53	0.20
	0.5 to 1.0	11.96	14.36	-0.70
	1.0 to 1.5	15.50	17.28	-0.85
	1.5 to 2.0	12.67	16.77	-1.42
SI Region	0 to 0.5	4.43	6.32	-0.55
	0.5 to 1.0	7.73	6.90	0.26
	1.0 to 1.5	3.74	4.44	-0.39
	1.5 to 2.0	5.20	6.35	-0.67
Intermediate	0 to 0.5	28.38	21.94	1.04
	0.5 to 1.0	23.12	22.95	0.03
	1.0 to 1.5	17.75	21.24	-0.51
	1.5 to 2.0	16.99	18.75	-0.32
ID Region	0 to 0.5	7.31	7.28	0.01
	0.5 to 1.0	3.84	6.99	-1.23
	1.0 to 1.5	6.92	6.45	0.22
	1.5 to 2.0	6.56	6.96	-0.16
Deep	0 to 0.5	25.11	29.64	-0.67
	0.5 to 1.0	28.11	33.09	-0.95
	1.0 to 1.5	26.84	31.62	-1.10
	1.5 to 2.0	27.34	27.94	-0.16
All Layers	0 to 0.5	81.69	80.70	0.06
	0.5 to 1.0	74.77	84.28	-0.59
	1.0 to 1.5	70.74	81.02	-0.81
	1.5 to 2.0	68.76	76.77	-0.76
Superficial	All	12.39	16.18	-1.05
SI Region	All	7.78	5.94	0.65
Intermediate	All	15.84	22.22	-0.93
ID Region	All	8.23	6.75	0.60
Deep	All	26.12	31.07	-0.79
All Layers	All	70.37	82.17	-0.75

Supplementary Table S2. The ratios of cells per unbranched vessel segment and proportion of the total cell population

Vascular Region	Cell Type	Ratio	%
Superficial	GCL	35	0.0648
	INL*	419	0.1347
	Amacrine	168	0.1349
	Bipolar	176	0.1346
	Horizontal	13	0.1347
	Cone	51	0.0647
	Rod	1758	0.0646
SI Region	GCL	97	0.1802
	INL*	1137	0.3653
	Amacrine	454	0.3641
	Bipolar	479	0.3661
	Horizontal	34	0.3653
	Cone	142	0.1808
	Rod	4936	0.1813
Intermediate	GCL	21	0.0392
	INL*	226	0.0726
	Amacrine	91	0.0727
	Bipolar	95	0.0725
	Horizontal	7	0.0726
	Cone	31	0.0394
	Rod	1072	0.0394
ID Region	GCL	75	0.1400
	INL*	865	0.2780
	Amacrine	345	0.2771
	Bipolar	363	0.2776
	Horizontal	26	0.2780
	Cone	110	0.1401
	Rod	3817	0.1402
Deep	GCL	14	0.0266
	INL*	165	0.0530
	Amacrine	66	0.0530
	Bipolar	69	0.0529
	Horizontal	5	0.0530
	Cone	21	0.0266
	Rod	725	0.0266

*Inner nuclear layer cells that were not distinguished, which included amacrine, bipolar, horizontal, and nonspecific cells

Supplementary Table S3. Two-way ANOVA results for each the entire vessel density and each trilaminar layer

Vascular Region	Source of Variation	Variation (%)	SS	DF	MS	F (DFn, DFd)	P value
All	Eccentricity x Age	6.32	717	12	59.75	F (12, 30) = 0.7017	0.737
	Eccentricity	2.345	266.1	3	88.69	F (3, 30) = 1.042	0.388
	Age	9.074	1029	4	257.3	F (4, 10) = 0.3881	0.813
	**Mouse**	**58.45**	**6631**	**10**	**663.1**	**F (10, 30) = 7.788**	**<0.0001***
Superficial	Eccentricity x Age	16.55	120.6	12	10.05	F (12, 30) = 1.251	0.297
	**Eccentricity**	**10.15**	**73.91**	**3**	**24.64**	**F (3, 30) = 3.067**	**0.043***
	Age	7.517	54.76	4	13.69	F (4, 10) = 0.5814	0.683
	**Mouse**	**32.32**	**235.4**	**10**	**23.54**	**F (10, 30) = 2.931**	**0.011***
SI Region	Eccentricity x Age	14.47	65.12	12	5.427	F (12, 30) = 1.103	0.393
	**Eccentricity**	**11.41**	**51.36**	**3**	**17.12**	**F (3, 30) = 3.480**	**0.028***
	Age	3.303	14.87	4	3.718	F (4, 10) = 0.2176	0.923
	**Mouse**	**37.95**	**170.8**	**10**	**17.08**	**F (10, 30) = 3.473**	**0.004***
Intermediate	Eccentricity x Age	12.09	275.3	12	22.94	F (12, 30) = 1.063	0.423
	Eccentricity	4.723	107.5	3	35.85	F (3, 30) = 1.661	0.197
	Age	12.86	292.8	4	73.2	F (4, 10) = 0.7978	0.553
	Mouse	40.29	917.6	10	91.76	F (10, 30) = 4.250	**0.001***
ID Region	Eccentricity x Age	13.17	43.29	12	3.607	F (12, 30) = 0.6978	0.741
	Eccentricity	1.237	4.065	3	1.355	F (3, 30) = 0.2621	0.852
	Age	2.02	6.638	4	1.659	F (4, 10) = 0.1404	0.963
	**Mouse**	**35.96**	**118.2**	**10**	**11.82**	**F (10, 30) = 2.286**	**0.039***
Deep	Eccentricity x Age	7.032	122.1	12	10.18	F (12, 30) = 0.6853	0.752
	**Eccentricity**	**11.57**	**201**	**3**	**67**	**F (3, 30) = 4.511**	**0.01***
	Age	11.64	202.2	4	50.56	F (4, 10) = 0.6847	0.619
	**Mouse**	**42.51**	**738.4**	**10**	**73.84**	**F (10, 30) = 4.971**	**0.0003***

*Significant results

Supplementary Table S4. Three-way ANOVA results for vessel density

Source of Variation	Variation (%)	SS	DF	MS	F (DFn, DFd)	P value
**Trilaminar Layer**	**85.29**	**6418**	**4**	**1604**	**F (4, 64) = 226.4**	**<0.0001***
Age	0.1521	11.45	1	11.45	F (1, 16) = 0.4983	0.490
Eccentricity	0.7495	56.39	1	56.39	F (1, 16) = 2.455	0.137
Trilaminar Layer x Age	0.6066	45.64	4	11.41	F (4, 64) = 1.610	0.182
**Trilaminar Layer x Eccentricity**	**1.585**	**119.3**	**4**	**29.82**	**F (4, 64) = 4.208**	**0.004***
Age x Eccentricity	0.3328	25.04	1	25.04	F (1, 16) = 1.090	0.312
Trilaminar Layer x Age x Eccentricity	0.3661	27.55	4	6.887	F (4, 64) = 0.9717	0.429
Mouse	4.885	367.5	16	22.97		

*Significant results

Supplementary Table S5. Three-way ANOVA multiple comparisons for each layer

Vascular Region	Tukey's multiple comparisons test	Mean Diff.	95.00% CI of diff.	P Value
Superficial	Young Adult: Central vs Peripheral Retina	-0.4511	-7.878 to 6.975	>0.9999
	Middle Aged: Central vs Peripheral Retina	-2.211	-9.637 to 5.216	0.9999
	Central Retina: Young Adult vs Middle Aged	2.271	-5.155 to 9.698	0.9998
	Peripheral Retinal: Young Adult vs Middle Aged	0.5119	-6.915 to 7.939	>0.9999
SI Region	Young Adult: Central vs Peripheral Retina	-0.7041	-8.131 to 6.723	>0.9999
	Middle Aged: Central vs Peripheral Retina	2.942	-4.485 to 10.37	0.995
	Central Retina: Young Adult vs Middle Aged	-2.265	-9.691 to 5.162	0.9999
	Peripheral Retinal: Young Adult vs Middle Aged	1.381	-6.045 to 8.808	>0.9999
Intermediate	Young Adult: Central vs Peripheral Retina	2.911	-4.515 to 10.34	0.9959
	Middle Aged: Central vs Peripheral Retina	7.185	-0.2412 to 14.61	0.070
	Central Retina: Young Adult vs Middle Aged	-3.126	-10.55 to 4.301	0.991
	Peripheral Retinal: Young Adult vs Middle Aged	1.148	-6.278 to 8.575	>0.9999
ID Region	Young Adult: Central vs Peripheral Retina	-0.8235	-8.250 to 6.603	>0.9999
	Middle Aged: Central vs Peripheral Retina	1.025	-6.402 to 8.452	>0.9999
	Central Retina: Young Adult vs Middle Aged	-0.3205	-7.747 to 7.106	>0.9999
	Peripheral Retinal: Young Adult vs Middle Aged	1.528	-5.899 to 8.954	>0.9999
Deep	Young Adult: Central vs Peripheral Retina	1.573	-5.854 to 8.999	>0.9999
	Middle Aged: Central vs Peripheral Retina	3.572	-3.854 to 11.00	0.964
	Central Retina: Young Adult vs Middle Aged	1.819	-5.608 to 9.245	>0.9999
	Peripheral Retinal: Young Adult vs Middle Aged	3.818	-3.608 to 11.24	0.934
